# News and Notes

**Published:** 1903-07

**Authors:** 


### *NEWS AND NOTES.*

---

CHOLERA has reappeared at Manila and in the archipelago, and is increasing with the return of the hot season.

---

THE *Philadelphia Medical Journal* has passed under control of the *New York Medical Journal* and the publication office has been moved to New York.

---

SIR FREDERICK TREVES, who was an examiner in medicine in the University of Aberdeen nineteen years ago, received the honorary degree of LL.D. April 8 last.

---

BETWEEN December 27, 1902, and April 24, 1903, 15,919 cases of smallpox with 441 deaths have occurred in the United States, as compared with 29,666 cases with 882 deaths for the same period last year.—*Exchange.*

---

DR. H. H. RODMAN, of New York, who was surgeon of the hospital ship *Maine,* has been presented by the British War Office with the war medal and clasp for the South African war, and also with the medal for the China war.

---

ANTI-VACCINATIONIST DIES OF SMALLPOX.—Charles Stevens, secretary of the Anti-vaccination League of Minneapolis, died from smallpox April 15. He had frequently denounced vaccination as inefficacious and a barbarous practice.

---

AMERICAN physicians and dentists will now be on the same standing in Turkey as are those in France. They have heretofore been refused examination and could not practice. They will now be allowed to practice if they present good diplomas and pass the examination. This is the result of pressure brought to bear on the Sultan by President Roosevelt and U. S. Minister Leishman. —*Exchange.*

---

IT is announced that Prof. Joseph Seegen proposes to offer a prize under the auspices of the Imperial Academy of Sciences in Vienna for the best answer to the following question: "Is any part of the nitrogen of the albuminates which have undergone metabolism in the animal body eliminated either by the lungs or by the skin in a gaseous form?" The prize offered amounts to about $1,000, and essays may be written in German, French or English, and must be sent before February 1, 1904.—*Philadelphia Medical Journal.*

---

AMERICAN ACADEMY OF MEDICINE.—At the meeting held in Washington, D. C., May 11 and 12, the following officers were elected for the ensuing year: President, Dr. John B. Roberts, of Philadelphia; vice-presidents, Drs. T. B. Davis of Pittsburg, J. H. McBride of Pasadena, Cal., J. P. Searcy of Tuscaloosa, Ala., and S. A. Knopf, of New York; Secretary, Dr. Charles McIntre, of Easton, Pa.; assistant secretary, Dr. A. R. Craig, of Columbia, Pa.; treasurer, Dr. E. A. Green, of Easton, Pa. The next meeting will be held at Atlantic City, N. J., June 11 to 13, 1904.

---

THE sale of cocain in Minnesota is to be regulated under a bill recently introduced in the lower house of the legislature of Minnesota. Under this bill cocain may be sold at retail only by pharmacists, and at wholesale only to pharmacists. Druggists may sell cocain only on the written prescription of a physician or dentist, and such prescription may be filled only once. It is also provided that any patent medicine, catarrh powder or remedy, snuff, cream, or other mixture containing cocain, must have a label stating that fact in the English language. A violation of the act is made a misdemeanor, punishable by a fine between $50 and $100, or imprisonment from thirty days to one year. One half of all such penalties shall go to the State Pharmacy Board, and one half to the county school fund.—*New York Medical Journal.*

---

JUNE 22, 1903.

*To the Members of the Medical Profession :*

At a conference of the officers and Advisory Committee of the American Congress on Tuberculosis, held in New Orleans, May 7, some important changes were made in the plans as previously announced.

The previous plans of the Council to hold the Congress in St. Louis, in 1904, were changed, many considerations favoring Washington, D. C., as the place of meeting. A change of time of meeting was also made to April 4, 5, and 6, 1905.

As there is to be an International Congress on Tuberculosis, at Paris, in 1904, it was deemed possible that some foreign delegates might be prevented from attending the Washington meeting on that account. The plan and scope of the American Congress being in reality international, the postponement of the meeting to the spring of 1905 will give the management ample time for perfecting details upon which the success of a Congress largely depends.

One committee has been already arranged to have charge of the Section on Pathology and Bacteriology, as follows: Dr. Simon Flexner, chairman; Dr. William H. Welch, Dr. George J. Adami, Dr. Thebald Smith, and Dr. F. F. Wesbrook. Committees in charge of other sections or departments will be announced later.

Dr. George Brown, of Atlanta, Ga., is practically the Executive Officer of the Congress, and all who desire to present papers before the Congress should apply to him. As there seems still to be a doubt in the minds of many physicians concerning the result of the vote of the New York meeting, which in 1902 adopted a new and definite plan for the next Congress, we beg to assure our readers that the new plan is being followed in both letter and spirit by Dr. Brown and the other officers elected at that meeting.

Any circulars or communications purporting to be in the interests of the American Congress on Tuberculosis, which do not appear over the name of Dr. George Brown, as Secretary, do not relate to the Congress which was arranged last year, and the organization of which has already so far advanced as to insure its success from every point of view. DANIEL LEWIS, M.D.,
President The American Congress on Tuberculosis.

---

### SURGICAL HINTS.

In very large foul sores the application of a charcoal poultice, after thorough preliminary irrigation, is often of great value.

No laparotomy patient will suffer very severely from thirst if given an enema of a pint of warm saline solution every three or four hours.

In a case of amputation of the uvula a neurasthenic patient for some time presented most alarming symptoms of dyspnea and partial asphyxiation.

If a patient has an attack of faintness while sitting in a chair, cause him to bend forward for a minute with his head nearly between his knees. This causes a marked tendency to cerebral congestion, and is often quicker and more convenient than pulling him to the floor and letting him lie flat on his back.

If possible, whenever you have to make an incision in the scalp, keep the blade of your knife in a direction parallel to that of the growth of the hair. For instance, at the back of the head, a transverse incision should be obliquely through the scalp, as this will cut through the smallest possible number of hair roots and cause the smallest amount of scarring.

The hypodermic injection of brandy, whisky or sulphuric ether is one of the best methods of combating intense shock and collapse, but the surgeon should always remember to inject them deep into the muscles, as they may cause sloughing of the skin if injected beneath it, and they should not be introduced in the neighborhood of any important nerve, as they have been known to cause paralysis or neuritis.—*International Journal of Surgery.*

